# Intracranial angioleiomyoma: a case series of seven patients and review of the literature

**DOI:** 10.1007/s11060-024-04734-y

**Published:** 2024-06-06

**Authors:** Meltem Ivren, Asan Cherkezov, David Reuss, Daniel Haux, Christel Herold-Mende, Alexander Mohr, Sandro M. Krieg, Andreas Unterberg, Alexander Younsi

**Affiliations:** 1 Department of Neurosurgery, Heidelberg University, Im Neuenheimer Feld 400, 69120 Heidelberg, Germany; 2 Department of Neuopathology, Heidelberg University, Im Neuenheimer Feld 672, Heidelberg, Germany; 3 Department of Neuroradiology, Heidelberg University, Im Neuenheimer Feld 400, Heidelberg, Germany; 4 Department of Neuroradiology, Klinikum Darmstadt, Grafenstraße 9, Darmstadt, Germany

**Keywords:** Clinical presentation, Differential diagnosis, Intracranial angioleiomyoma, Smooth muscle actin, Case series

## Abstract

**Purpose:**

Angioleiomyoma, predominantly arising from the extremities, is a benign soft tissue tumor. Reports on its intracranial location are rare. We assessed clinical, radiological, and pathological features of intracranial angioleiomyoma (iALM) treated at our neurosurgical institution.

**Methods:**

We consecutively enrolled all patients with neuropathologically confirmed iALM treated at a single neurosurgical institution between 2013 and 2021. Clinical and imaging data were collected, and histological tissue sections were analyzed. A review of the literature on iALM was conducted.

**Results:**

Seven patients with iALM (four female) with a median age of 45 years (range: 32–76 years) were identified. In three cases, the lesion was found incidentally. In magnetic resonance imaging (MRI), all tumors were hypo- to isointense on T1-weighted, hyperintense on T2-weighted sequences, and gadolinium-enhancing. A strong FLAIR signal was seen in six patients. Surgery consisted of gross total resection in all cases without perioperative complications. Neuropathological staining was positive for smooth muscle actin (SMA) in all lesions. Mature smooth muscle cells arranged around blood vessels were typically observed. The Ki-67 index was ≤ 3%. The patients were discharged after a median of 6 days (range: 4–9 days). During a median follow-up time of 14 months (range: 4–41 months), no tumor recurrence occurred. In the current literature, 42 additional cases of iALM were identified.

**Conclusion:**

Intracranial angioleiomyoma is a benign soft tissue tumor treated by gross total resection. Tumor morphology and positive staining for SMA lead to the neuropathological diagnosis.

**Supplementary Information:**

The online version contains supplementary material available at 10.1007/s11060-024-04734-y.

## Introduction

Angioleiomyoma (or angiomyoma/ vascular leiomyoma [1]) is a benign soft tissue tumor with smooth muscle and vascular components [2–5]. It predominantly presents in the middle-aged population, often as a subcutaneous lump in the extremities [4, 6, 7]. Angioleiomyomas account for 4–5% of soft tissue tumors and are subdivided into three histological subtypes: solid, venous, and cavernous [8]. In 1984, Hachisuga et al. presented a large series of 562 angioleiomyoma cases with a predominance of the female gender [9]. The most prevalent locations were the extremities (89%), the head (8.5%) and the trunk (2.5%) [9]. Ten years later, Lach et al. published the first case report about an intracranial angioleiomyoma (iALM) in the right parietal lobe [10]. To date, 42 cases of iALM have been reported. In the current study, we present seven new cases of iALM and convey a literature review on its rare intracranial location.

## Methods

### Patient data

We retrospectively included all iALM patients treated at our neurosurgical institution from 2013 to 2021. Patients with extracranial/ strictly intraosseous manifestations were excluded (Online Resource 1). Neurological examination was performed on patient admission, discharge, and different follow-up time points. Data regarding patients’ demographics, neurological condition, and surgical and histologic characteristics were extracted from our institutions’ electronic database. Pre- and postoperative MRI data were jointly evaluated by experienced neurosurgeons and neuroradiologists. This analysis of iALM patients was approved by the local Ethics Committee and was conducted in accordance with the PROCESS guidelines [11].

### Pathological examination

All tissue samples were stained with hematoxylin and eosin. Immunohistochemical staining was added during diagnostics and varied in each case over the years. However, specific staining for smooth muscle actin (a-SMA), CD31, and CD34 were performed invariably. For this study, we microscopically classified the tumors according to the pre-described subtypes: cavernous, venous, and solid [12].

### Literature review

The literature review was performed using the Pubmed library. The terms used for the search engine were “intracranial angioleiomyoma”, “intracranial angiomyoma”, and “intracranial vascular leiomyoma”. All publications written in English, starting from 1994, were considered.

## Results

### Patient demographics

Seven iALM patients with a median age of 45 (range: 32–76) years were surgically treated at our neurosurgical institution (Tables 1 and 2). The female gender was predominant (*n* = 4; 57%). While the lesions were found incidentally in three patients, four patients suffered from tumor-associated headaches (*n* = 3), visual impairment (*n* = 1), and gait disturbance with hypesthesia of the face (*n* = 1).

**Table 1:** Demographic, clinical, and treatment characteristics of the seven intracranial angioleiomyoma patients

Case	Age	Sex	Symptoms	Symp-tom duration	Tumor localization	Size [mm]	EOR	Differential diagnosis	Re-currence	Follow-up time [months]
1	32	f	Incidental finding		Cerebral falx	10	GTR	ALM, MGM	No	13
2	36	m	Incidental finding		Left cerebellar	12	GTR	MGM, HPC, Cavernoma	No	15
3	45	f	Incidental finding		Right parieto-occipital	14	GTR	MGM	No	27
4	43	f	Right frontal headaches	8 weeks	Right frontal	15	GTR	MGM, HPC	No	4
5	56	f	Visual impairment	N/A	Apex orbitae left	15	GTR	NEU, MGM	No	6
6	61	m	Headaches around the right eye	6 months	Right tentorial with supra- and infratentorial portion	28	GTR	MGM	No	41
7	76	m	Gait disturbance, hypesthesia right face	N/A	Right cerebellar	35	GTR	MGM	No	3

EOR = extent of resection, GTR = gross total resection, ALM = angioleiomyoma; MGM = meningioma, HPC = hemangiopericytoma, NEU = neurinoma, N/A = not available

**Table 2:** Radiographic and histopathological characteristics

Case	T1	T2	FLAIR	T1 CE	Positive	Negative	Macroscopy	Subtype
1	Iso	Hyper	Hyper (strong)	Partially enhancing	SMA, Calponin	HMB45, Ki-67 1%	Reddish-livid, tenacious	Cavernous
2	Iso	Hyper	Hyper (strong)	Strongly enhancing	CD31 (vessels), SMA, Desmin, Caldesmon	EMA, S100, somatostatin receptor, ERG, Ki-67: 2%	Greyish-brown, tenacious	Solid + cavernous
3	Iso	Hyper	Hyper (strong)	Homogenously enhancing	Aktin-SMA, CD 31, CD34	S100, somatostatin-receptor, Ki 67: 1–3%	Tenacious-solid, brown	Solid
4	Iso	Hyper	Hyper (strong)	Homogenously enhancing	EMA (partially), CD 31(vessels), Aktin-SMA, Desmin (partially)	K-67: 3%	Abundant blood supply, tenacious consistency	Solid
5	Hypo	Hyper	Hyper (strong)	Homogenously enhancing	AE1/3 (paritally), CD31, CD34, Vimentin, Aktin-SMA, Ki 67: 2–3%, LCA (some leucocytes positive)	NFP, S100, STAT6, D2-40(Podoplanin), Syanptophysin, Chromogranin	Abundant blood supply, venous bleeding, tenacious consistency, reddish-brown	solid
6	Iso	Hyper	Hyper (strong)	Strongly enhancing	EMA (partially), Kollagen IV, CD 31, CD34, Aktin-SMA, Desmin (Partially)	Stat6 Ki67: 3%	Tenacious, many feeding vessels, abundant blood supply, reddish-white	Solid + cavernous
7	Hypo	Hyper	Hyper	Inhomogenously enhancing	CD31, CD34, SMA	EMA; EBV, Desmin, Ki-67 < 1%	Tenacious, reddish-grey	Cavernous

T1 = T1 sequence, T2 = T2 sequence, FLAIR = fluid-attenuated inversion recovery sequence, T1 CE = T1 with gadolinium injection, Iso/Hypo/Hyper = iso-/hypo-/hyperintense

### Imaging findings

**Fig. 1:**
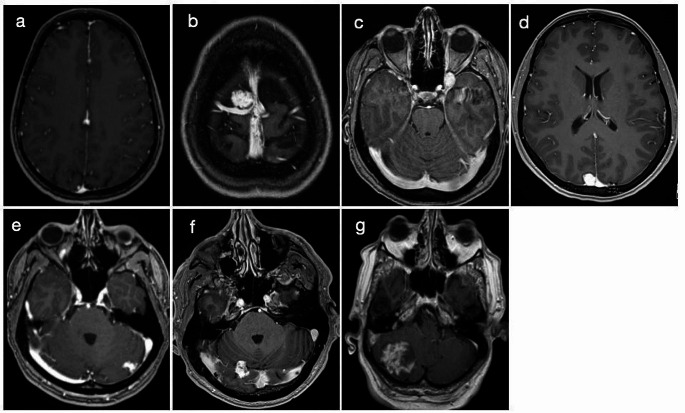
Case series of seven intracranial angioleiomyoma patients Preoperative T1 axial MRI sequences with gadolinium enhancement of every patient showing the seven angioleiomyoma cases in the following locations: (**a**) cerebral falx (illustrated case). (**b**) right superior frontal gyrus. (**c**) left orbital apex. (**d**) parietooccipital. (**e**) left cerebellar. (**f**) right tentorial with supra- and infratentorial portion. (**g**) right cerebellar

On T1-weighted MRI, the lesions presented mostly iso- (*n* = 5) or hypointense (*n* = 2). All were hyperintense on T2- sequences; a powerful signal on the FLAIR sequence was present among six cases. No perilesional edema was found. All lesions were fully or partially gadolinium-enhancing. Two lesions were located strictly infratentorial and one tumor had a supra- and infratentorial expansion. The remaining four iALMs were strictly supratentorial, located in the cerebral falx (illustrated case), the superior frontal gyrus, the left orbital apex, and the parietooccipital lobe (Fig. 1). All lesions were defined as being extraaxial. The mean lesion size was 18.4 +/- 9.3 mm (range: 10–35 mm). Initially, meningioma was the suspected diagnosis in all cases. In the illustrative case, the suspected diagnosis was changed to iALM preoperatively. In another case, cavernoma was suspected due to a large vein draining into the left transverse sinus. Other primary differential diagnoses were hemangiopericytoma (*n* = 2) and neurinoma (*n* = 1).

### Surgery, postoperative course, and follow-up

A gross total resection was achieved in all cases without intraoperative complications. All surgeons described tumor consistency as tenacious; a reddish or brown color was reported in six cases. An apparent abundant blood supply was mentioned three times. Postoperatively, the patient with the tumor in the orbital apex suffered from a new abducens nerve palsy that fully recovered at follow-up. No other postoperative neurological deficits occurred, and the patients were discharged home after a median of 6 days (range: 4–7 days). At follow-up after a median of 14 months (range: 4–41 months), no recurrence or clinical deterioration was seen.

#### Histopathological findings

All lesions were well circumscribed without infiltration of surrounding tissue. No nuclear atypia or mitotic figures were present. Ki-67 proliferation index was ≤ 3%. Hematoxylin and eosin staining revealed well-differentiated monomorphic spindle-shaped smooth muscle cells centered around medium-sized blood vessels. The vessel walls were positive for CD31 and CD34 staining in all cases. The smooth muscle cells were strongly positive for actin-SMA. Two cases were classified as cavernous subtypes; three were classified as solid subtypes. In two cases, aspects of both cavernous and solid subtypes were seen. None of the cases was classified as the venous subtype.

### Illustrative case

A 32-year-old woman, previously diagnosed with an intracranial lesion, presented to our neurosurgical department with unspecific symptoms, such as intermittent dizziness. She suffered from anxiety, panic disorder and Hashimoto thyroiditis. The initial MRI showed a homogenously gadolinium-enhancing lesion of 5–6 mm in the cerebral falx. Due to the suspicion of meningioma, the small size, and the absence of neurological deficits, a watch-and-wait strategy was decided. Within the next five years, there was a steady growth to a maximal diameter of 10 mm. Despite considerations for continued follow-up, the patient’s concerns led to surgical intervention, taking the consistent tumor growth into account.

**Fig. 2:**
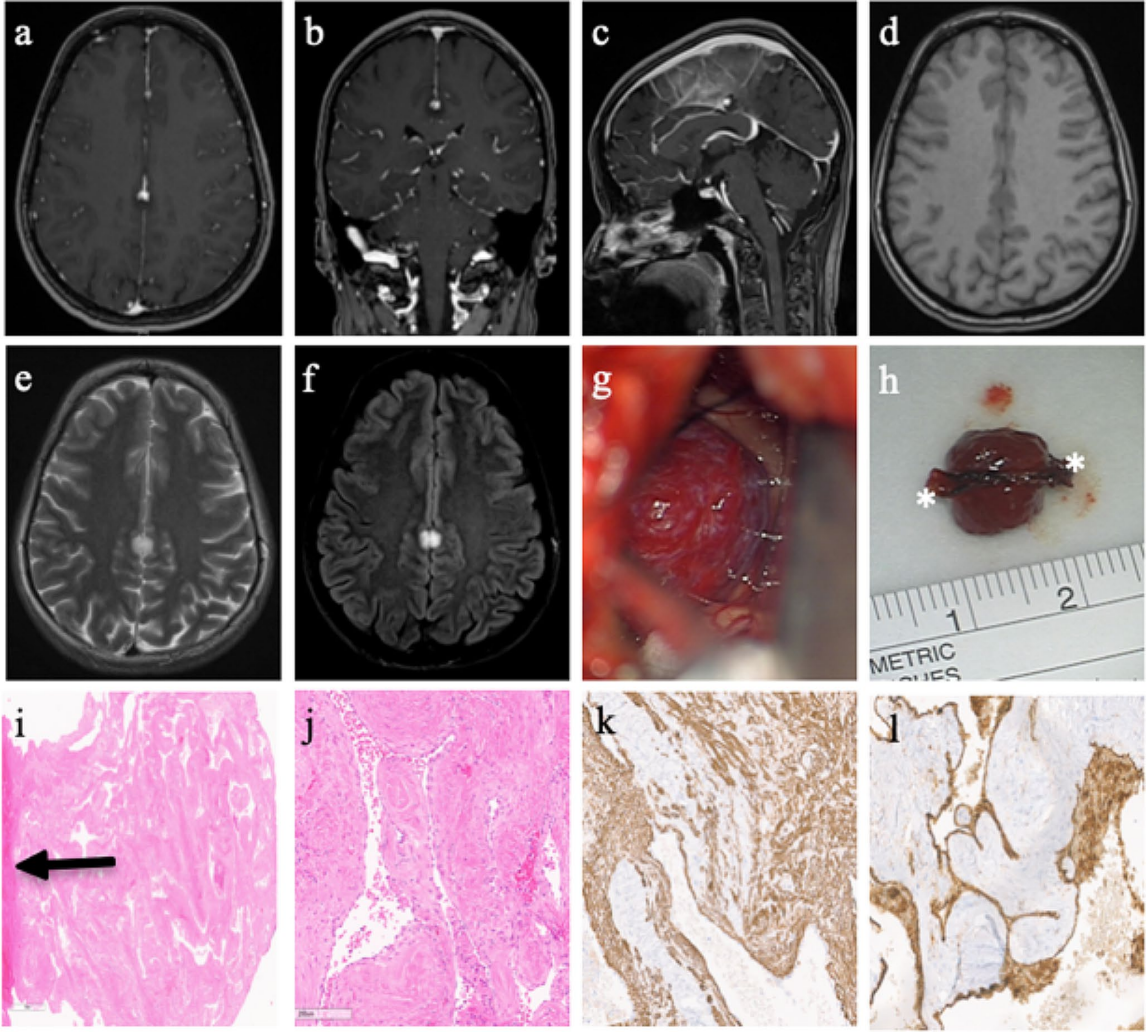
Illustrative case of an angioleiomyoma of the cerebral falx **a-c**) Axial, coronal and sagittal T1 MRI sequences showing a small, partly gadolinium-enhancing lesion of the cerebral flax, **d-f**) T1, T2 and FLAIR axial MRI sequences showing T1 isointensity, T2 hyperintensity and a strong FLAIR signal, **g**) Preparation of the lesion after gentle traction of parasagittal cortical tissue. Bulging vessels can be seen on the tumor surface. **h**) Macroscopic presentation of the 10 mm measuring livid-reddish lesion with attachment to the falx (stars). **i**) Hematoxylin-eosin staining showing clear tumor boarders and the corresponding dural attachment (arrow). **j**) Close-up of hematoxylin-eosin staining showing a cavernous subtype tumor with variably dilated vascular channels and smooth muscle walls. **k**) Anti-SMA (smooth muscle actin) staining showing a strong signal of the smooth muscle cells, **l**) anti-CD31-staining for identification of endothelial layer of the vessels

Preoperative imaging showed a 10 mm, symmetrically arranged mass around the cerebral falx with an iso-intense signal on T1, a hyperintense signal on T2, and a strong hyperintense signal on the FLAIR sequence (Fig. 2). The lesion showed partial gadolinium enhancement in the anterior portion only. Compared to prior imaging, there was a decrease in contrast enhancement. Due to the high FLAIR signal and the changing behavior of contrast enhancement, the rare differential diagnosis of angioleiomyoma was suggested.

Tumor resection was conducted by a frontoparietal craniotomy over the sinus using neuronavigation. After gentle retraction of the parasagittal cortical zone, the lesion was visualized: It appeared reddish-livid with bulging vessels covering the surface. The tumor was not adherent to surrounding cortical tissue and did not involve the pericallosal arteries. Eventually, the lesion was excised en bloc with a safety margin of 1–2 mm. The postoperative course was uneventful, and the patient was discharged four days after surgery. Postoperative MRI after four months showed no recurrence. Yet, the patient presented twice at the emergency department with another episode of dizziness, headaches, and a pressure feeling in both eyes nine and 13 months postoperatively. Both times, clinical examination and brain imaging remained unremarkable.

Neuropathological examination of hematoxylin-eosin staining revealed numerous dilated vessels with monomorphic spindle-shaped cells in between, the nuclei being oval-shaped without prominent nucleoli. Neither mitotic activity (examination of 10 high-power fields) nor necrosis was seen in the tissue sample. The Ki-67 labeling index was 1%. Immunohistochemistry showed positive staining for smooth muscle actin (SMA) and Calponin. HMB45 was negative. Endothelial cells of the vessel walls showed positive staining for CD31.

### Review of the literature

In 28 publications from 1994 to 2023, 42 cases could be identified (Table 3). Li et al. [13] (8 cases), He et al. [14] (4 cases) and Sun et al. [15] (3 cases) presented case series. Two more publications presented two cases each (see Online Resource 2); the rest were case reports of single cases. Overall, the median age was 47 years (range: 12–77 years), and 68.3% were male. Headaches were present in 52.4%. The most common tumor locations were the cavernous sinus/ sellar region (35.7%), followed by the posterior fossa (26.2%; cerebellar/ tentorial: *n* = 8, inner auditory meatus/ cerebellopontine angle: *n* = 3), cerebral falx (11.9%) and the temporal lobe (9.5%). The remaining seven cases included two basal ganglia manifestations (4.9%), interestingly both presenting as multicentric lesions. 30 lesions were described to be located extraaxial and ten intraaxial.

**Table 3:** Clinical and diagnostic parameters from the literature and the present series

	Prior studies (*n* = 42*)	Present series (*n* = 7)	Overall (*n* = 49*)
Median age (range)	47 (12–77)	45 (32–76)	47 (12–77)
Sex (female)	31.7%	57.1%	35.4%
Clinical presentation			
Headache	22 (52.4%)	2 (28.6%)	24 (49.0%)
Seizure	7 (16.7%)	0 (0%)	7 (14.3%)
Focal deficit^a^	27 (64.3%)	2 (28.6%)	29 (59.2%)
Tumor location			
Cavernous sinus/ Sellar Posterior fossa^b^ Cerebral falx Other	15 (35.7%) 11(26.2%) 5 (11.9%) 11 (26.2%)	0 (0%) 3 (42.9%) 1 (14.3%) 3 (42.9%)	15 (30.6%) 14(28.6%) 6 (12.2%) 14 (28.6%)
Mean size [mm] (range)	31.0 (7.0–77.0)	18.4 (10.0–35.0)	29.0 (7.0–77.0)
MRI characteristics			
T1 iso/hypo/hyper (%)	13/19/0 (40.6/59.4/0.0)	5/2/0 (71.4/28.6/0.0)	18/ 21/ 0 (46.2/53.8/0.0)
T2 iso/hypo/ hyper (%)	2/1/31 (5.9/2.9/91.2)	0/0/7 (0/0/100)	2/1/38 (4.9/2.4/92.7)
Contrast enhancing yes/no (%)	33/0 (100%/0%)	7/0 (100%/0%)	40 (100%/0%)
Homogenous/ nonhomogenous/ progressive enhancement^c^ (%)	9/10/13 (28.1/31.3/40.6)	3/4/7 (42.9/57.1/0)	12/14/13 (30.8/35.9/33.3)
Differential diagnosis			
MGM-HPC/ SCHW-NEU/ CavHem-VM/ Other (%)	15/3/6/6 (50.0/10.0/20.0 /20.0)	4/1/0/1 (66.7%/16.7%/ 0/16.7%)	20/4/6/7 (54.1%/10.8%/ 16.2%/18.9%)
GTR/STR/Other (%)	34/6/2 (81.0/14.3/4.8)	7/0/0 (100/0/0)	41/6/2 (83.7/12.2/4.1)
SMA positive (% )	40 (100)	7 (100)	47 (100)
Recurrence (%)	1 (3.1)	0 (0)	1(2.6%)
Median follow-up time [months] (range)	20 (0.5–72)	14 (4–41)	19 (5–72)

All individual cases from the literature review [1–7, 10, 13–16, 18, 19, 21–34] are listed separately in a supplementary table (Online Resource 2)

iso/hypo/hyper = iso-/ hypo-/ hyperintense on respective MRI sequence, MGM-HPC = meningioma/ hemangiopericytoma, SCHW-NEU = schwannoma/neurinoma, CavHem-VM = cavernous hemangioma/ vascular malformation, GTR/STR = gross total/ subtotal resection, SMA = smooth muscle actin

* Note that percentages are given with reference to the available data

a) Focal deficits not further specified as mostly due to tumor location

b) Including cerebellopontine angle and tentorial lesions

c) Note that progressive enhancement is time dependent and not strictly separable from homogenous/nonhomogenous enhancement. However, it is listed here separately to show how often it was reported

A gross total resection (GTR) could be achieved in 33 patients (81.0%). In general, other than new postoperative deficits in accordance with tumor location, the postoperative course was described to be uneventful. However, Sun et al. reported a fatal postoperative complication in a case with an intrasellar manifestation of iALM: the patient had bled from a pseudoaneurysm of the left cavernous ICA segment [15]. Ravikumar et al. reported on a multifocal basal ganglionic iALM patient who preoperatively died from septicemia [16]. In the other reported multifocal iALM case, published by Shinde et al., only one of the lesions was resected, and the patient developed dystonia, rigidity, and tremor in all limbs [17]. Lastly, Rubiu et al. interrupted resection because of heavy bleeding from the tumor [18].

The cavernous subtype was the most common out of 22 cases where histopathological subclassification was conducted (*n* = 18, 81%). The venous and the solid subtypes were each reported twice (9.1%). Histopathological staining was positive for smooth muscle actin (SMA) in 95.2%. In the remaining two cases, this information was not given.

After a median follow-up time of 20 months (range: 2 weeks − 72 months), one of the patients showed recurrence after subtotal resection, as reported in the second latest publication of Rubiu et al. [18]. This patient and one more case describe the only instances in which subtotal resection was combined with radiosurgery [1, 18]. No additional cases with adjuvant therapies were reported in the literature.

## Discussion

Angioleiomyoma is a benign soft tissue lesion developing from vascular smooth muscle cells. The 2020 WHO classification of soft tissue and bone tumors claims a morphological continuum between angioleiomyoma and myopericytoma [12]. An intracranial location is rare; nevertheless, intracranial angioleiomyoma are thought to arise from the dura mater near large venous or diploic vessels [13]. In this study, we presented a series of seven surgically treated patients, hence, to our knowledge, the second largest series so far. We reviewed the literature and collected 42 additional cases, representing the largest overview currently available.

### Clinical presentation

In our series, headaches were present in 29%. Despite the relatively small lesion size, the reported pain was well-circumscribed following tumor location. In the literature review, 52.4% were reported to suffer from headaches. Given the histopathological structure of angioleiomyoma, local ischemia due to smooth muscle contraction could be associated with the origin of pain [19, 20].

Of note, two more angioleiomyoma cases presented to our neurosurgical institution but were excluded due to tumor location: one patient noticed a preauricular palpable mass, which turned out to be an angioleiomyoma manifestation of the zygomatic branch of the facial nerve. The other patient with a strictly intraorbital retrobulbar angioleiomyoma presented with exophthalmos and was admitted to us by our ophthalmology department (Online Resource 1).

### Imaging and differential diagnosis

Li et al. have previously published a series of eight cases and suggested the cavernous sinus as a predilection site [13]. In our series, none of the lesions were located in the cavernous sinus. On the other hand, posterior fossa manifestations were seen relatively often. However, adding our cases to the 42 in the literature, the cavernous sinus remains the predilection site with 30.6%.

On MRI, all lesions in our series showed an iso- to hypointense signal on T1 and a hyperintense signal on the T2 as well as the FLAIR sequences in accordance with the literature: Within the available reported data, all iALM showed hypo- to isointensity on the T1 sequence (*n* = 32; hypointense: *n* = 19 (59.4%); isointense: *n* = 13 (40.6%)). Most showed hyperintensity on the T2 sequence (hyperintense: *n* = 31 (91.2%)). All but one of our cases showed a powerful hyperintense signal on the FLAIR sequence, which is most probably linked to the abundant blood supply of the lesions. A hyperintense signal on the DWI sequence should rather be attributed to T2 shine-through rather than diffusion restriction.

Gadolinium enhancement showed different behaviors, with some lesions enhancing homogeneously and others inhomogeneously. Partial enhancement in the form of a “flame-like” appearance has been described by some authors to be a specific radiographic feature for angioleiomyoma, often being referred to as progressive enhancement, describing enhancement arising centrally and spreading towards the outer tumor borders [13, 21]. Interestingly, the partial gadolinium enhancement in our illustrative case was the decisive reason to suggest angioleiomyoma as a differential diagnosis on preoperative imaging. In all other cases, meningioma was the most common suggested differential diagnosis, along with hemangiopericytoma and neurinoma. Of note, according to our literature review, our illustrative case is only the second case ever suspected to be an angioleiomyoma preoperatively.

The difficulty in identifying angioleiomyomas preoperatively underlines the need for a better understanding of the radiographical features. However, some distinctive features compared to other entitites have been reported: Meningiomas tend to show rapid maximal gadolinium enhancement and arterial blush rather than venous pooling in MR perfusion signal-time curves [13, 22]. Angiographically visualized artero-venous blush in the late venous phase in iALM is shown in one of the latest iALM publication [18]. In contrast, the dural attachment with a “dural tail sign” occur in meningioma and angioleiomyoma [13, 23]. Furthermore, calcification and perifocal edema on CT imaging might favor the differential diagnosis of meningioma over angioleiomyoma [23]. When located at the falx or tentorium, meningiomas mostly respect the dural wall and grow unilaterally. In contrast, angioleiomyoma seemingly originate from the center and then grow in both directions. As a further differential diagnosis, cavernous hemangioma tend to be hyperintense on T1 sequences and show a hemosiderin ring [13]. As angioleiomyomas can present with large feeding vessels, arteriovenous malformations are another differential diagnosis [10]. Of note, in one of our cases (Table 1), a large vein draining to the left transverse sinus was seen, and a cavernoma was suspected. Finally, pituitary adenoma, neurinoma, and dural metastases should be evaluated as further differential diagnoses depending on the integrity of the pituitary gland and stalk, on tumor location and appearance [10, 21].

### Histopathological findings

The tumors in our series were sharply circumscribed, benign tumors. All contained well-differentiated smooth muscle cells centered around medium-sized blood vessels. No signs of nuclear atypia or mitotic figures were seen. Two morphological subclasses (solid and cavernous) were identified. The cavernous subtype presents with dilated vascular channels with well-differentiated smooth muscle cells in the intervascular space. The solid subclass is represented by numerous slit-like vascular channels, the lumina sometimes being hardly identifiable. However, a clear-cut differentiation of the subtypes is not always possible. Hachisuga et al. saw a predominance of the solid subtype (66%) in their large series of 562 cases, with the vast majority of lesions being in the extremities (89%) [9]. To date, Li et al. presented the largest series of iALM (*n* = 8) and classified all their cases as cavernous subtypes [13]. Apart from localization, the solid subtype has been stated to occur more often in females, while the cavernous and venous subtypes are instead seen in male patients [22].

Immunohistochemical positivity for CD31 or CD34 helps identify the vessel endothelia. Actin-SMA is strongly positive in the smooth muscle compound, thicker than a normal vascular wall. Ki-67 proliferation index is low (≤ 3%). In accordance with the literature, none of our cases showed mitoses or nuclear atypia.

### Surgery and outcome

In our series, all patients received GTR without peri- or postoperative complications (one patient suffered from a transient abducens nerve palsy). Accordingly, recurrence during follow-up was not seen in any of our patients and only in one out of 42 cases reported in the literature.

Despite this benign behavior of angioleiomyomas regarding surgical resectability and recurrence, Sun et al. presented a case with postoperative fatal bleeding of an internal carotid pseudoaneurysm after resection of a cavernous sinus angioleiomyoma. They discussed a potential erosion of the ICA wall by the tumor and concluded a prior use of angiography might have been beneficial [15]. The preoperative use of DSA, possibly even with preoperative embolization, was also proposed by other authors, suggesting a better evaluation of tumor-feeding arteries or associated aneurysms as well as a reduction of intraoperative bleeding [13, 18, 24]. No DSA was performed before surgery in our case series, but no relevant intraoperative bleeding was reported either.

### Limitations

This is a retrospective case study with known drawbacks. Throughout the years 2013–2021, the conducted histopathological stainings varied. Though the second-largest case series in current literature, the low case numbers limit conclusions, drawn from a small overall cohort. Also, the literature review focused on English-language publications only. Lastly, the presented work is a single-center study bearing the risk for institutional bias - hence potentially affecting patient selection and surgical indications.

## Conclusion

Intracranial angioleiomyoma is a benign soft tissue lesion. Total resection can be considered curative without the need for adjuvant therapies. Radiologically, often confused for other extraaxial pathologies, positive staining of mature smooth muscle cells for SMA and distinct microscopical morphology lead to the correct histopathological diagnosis.

### Electronic supplementary material

Below is the link to the electronic supplementary material.

Supplementary Material 1: Online Resource 1, Two cases of extracranial angioleiomyoma of the head: Caption: Two cases with neurosurgically treated, but extracranially located angioleiomyoma. Axial T1 sequences after gadolinium injection (**a**) Retrobulbar angioleiomyoma. This patient presented with an exophthalmos without any visual deficit or pain. (**b**) Angioleiomyoma of the zygomatic branch of the facial nerve. The patient noticed a preauricular lump when going to the hairdresser. There was no accompanying pain or facial nerve deficit. In both patients, GTR was achieved without any perioperative complications. Both lesions were iso-/hypointense on T1 and hyperintense on T2 and FLAIR imaging. Histopathological findings were in accordance with the findings in our iALM series and the literature. Due to their extracranial location, the two cases presented here were excluded from the iALM series; Online Resource 2: Table[1-7, 10, 13-16, 18, 19, 21-34] Literature review of intracranial angioleiomyoma cases

## Data Availability

Most data generated or analysed during this study are included in this published article and the supplementary files. However, where necessary, further information is available from the corresponding author on reasonable request.

## Abbreviations

ADC: apparent diffusion coefficient
a-SMA/ SMA: smooth muscle actin
CavHem-VM: cavernous hemangioma/ vascular malformation
CT: computed tomography
DSA: digital subtraction angiography
DWI: diffusion weighted imaging
EOR: extent of resection
GTR: gross total resection
HPC: hemangiopericytoma
(i)ALM: (intracranial) angioleiomyoma
ICA: internal carotid artery
MGM: meningioma
N/A: not available
NEU: neurinoma
MRI: magnetic resonance imaging
STR: subtotal resection
SCHW: schwannoma

## Declarations

### Previous presentation

This material was partly presented as an oral abstract at the 73rd German Society of Neurosurgery congress in Cologne, Germany, 05/2022 and as a poster at the EANS congress in Barcelona, Spain, 09/2023.
