# Supplementary material for: Intracranial angioleiomyoma: a case series of seven patients and review of the literature
Source: J Neurooncol. 2024 Jun 6;169(2):399–408. doi: 10.1007/s11060-024-04734-y (PMC11341739; doi:10.1007/s11060-024-04734-y)
Supplement: Supplementary file 1 — Supplementary Material 1: Online Resource 1, Two cases of extracranial angioleiomyoma of the head: Caption: Two cases with neurosurgically treated, but extracranially located angioleiomyoma. Axial T1 sequences after gadolinium injection (a) Retrobulbar angioleiomyoma. This patient presented with an exophthalmos without any visual deficit or pain. (b) Angioleiomyoma of the zygomatic branch of the facial nerve. The patient noticed a preauricular lump when going to the hairdresser. There was no accompanying pain or facial nerve deficit. In both patients, GTR was achieved without any perioperative complications. Both lesions were iso-/hypointense on T1 and hyperintense on T2 and FLAIR imaging. Histopathological findings were in accordance with the findings in our iALM series and the literature. Due to their extracranial location, the two cases presented here were excluded from the iALM series; Online Resource 2: Table[1-7, 10, 13-16, 18, 19, 21-34] Literature review of intracranial angioleiomyoma cases [file 11060_2024_4734_MOESM1_ESM.pdf]

Supplementary Information (SI)

Article title: Intracranial angioleiomyoma: a case series of seven patients and review of the literature
Journal name: Journal of Neuro-Oncology
Author names: Meltem Ivren, MD, Asan Cherkezov, MD, David Reuss, MD PhD, Daniel Haux, MD, Christel Herold-Mende, PhD, Alexander Mohr, MD, Sandro M Krieg, MD PhD, Andreas Unterberg, MD PhD, Alexander Younsi, MD PhD
Correspondence
PD Dr. med. Alexander Younsi
Department of Neurosurgery, Heidelberg University,
Im Neuenheimer Feld 400, 69120 Heidelberg, Germany
E-mail: alexander.younsi@med.uni-heidelberg.de

Online Resource 1: Two cases of extracranial angioleiomyoma of the head

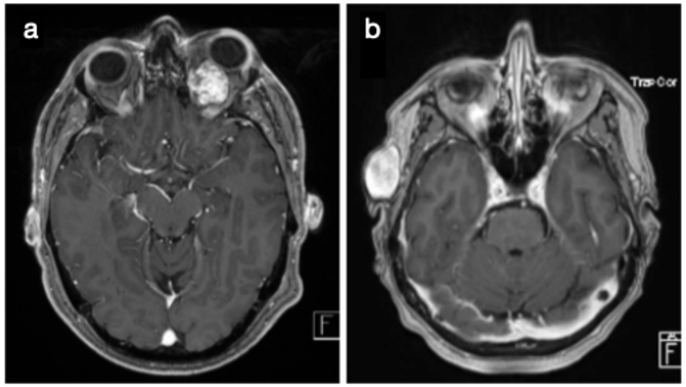

Two cases with neurosurgically treated, but extracranially located angioleiomyoma. Axial T1 sequences after gadolinium injection **a)** Retrobulbar angioleiomyoma. This patient presented with an exophthalmos without any visual deficit or pain. **b)** Angioleiomyoma of the zygomatic branch of the facial nerve. The patient noticed a preauricular lump when going to the hairdresser. There was no accompanying pain or facial nerve deficit. In both patients, GTR was achieved without any perioperative complications. Both lesions were iso-/hypointense on T1 and hyperintense on T2 and FLAIR imaging. Histopathological findings were in accordance with the findings in our iALM series and the literature. Due to their extracranial location, the two cases presented here were excluded from the iALM series.

Online resource 2: Literature review of intracranial angioleiomyoma cases

Case	Publication	Age	Sex	Symptoms + duration	Tumor localization	Suspected diagnosis	Tumor size (mm)	Treatment	MRI	CT	SMA posi-tive	Recurrence	Follow up (months)	Subtype
1	Lach et al. (1994) [17]	47	M	Gait disturbance, Right hand incoordination (14 months)	Right parietal lobe	Vascular malformation	27	GTR	N/A	Hypodense	yes	No	48	combination of subtypes
2	Ravikumar et al. (1996) [22]	12	F	Headache, diplopia, seizure, Left hemidystonia, visual deficit, hyporeflexia, apraxia of eye lid (2 months)	Right caudate nucleus + left globus pallidus	N/A	N/A	GTR of the right basal ganglionic lesion	N/A	Right basal ganglionic lesion: Hypodense<br>Left pallidal lesion: hyperdense	N/A	No	20	Solid
3	Kohan et al. (1997) [16]	N/A	N/A	Hypacusis + tinnitus	IAM/CPA		N/A	GTR	N/A	N/A	N/A	N/A	N/A	
4	Figueiredo et al. (2005) [10]	52	M	Headache, diplopia, visual deficit, facial numbness (24 months)	Cavernous sinus		60	GTR	T1 iso<br>T2 hyper<br>T1CE: hen	Hyperdense, hen	yes	N/A	N/A	
5	Karagama et al. (2005) [15]	47	F	Hearing loss left, vertigo (18 months)	Internal auditory meatus	Vestibularis schwannoma	10	GTR	T1CE: hen	?	yes	No	12	

6	Colnat-coulbois et al. (2008)[6]	50	M	Headache, trochlear nerve palsy	Left cavernous sinus			GTR	T1 iso T2 hyper T1CE: PE		yes	No	72	
7	Gasco et al. (2009) [11]	43	M	Headache, blurred vision, ataxia, dydiadochokinesia, left dysmetria (acute onset)	Left cerebellar	Meningeoma	44	GTR	T1 iso T2 hyper T1-CE: hen	isodense	yes	N/A	N/A	Venous
8	Chongxiao et al. (2009)[5]	50	M	Headache, seizure (6 months)	Cerebral falx		40	GTR	T1 hypo T2 hyper T1CE: hen	hyperdense hen	yes	No	18	
9	Pepper et al. (2010) [21]	13	F	Headache, hearing loss (12 months)	IAM	Vestibularis schwannoma	7	GTR	T1 iso T2 iso T1 CE: hen	Bone window available only	yes	No	6	
10	Xu et al. (2010) [29]	53	M	Headache (3 months), visual deficit, temporal hemianopsia (1 month)	Sella turcica	Pituitary apoplexy	<10	GTR	T1 iso T2 hyper	N/A	yes	N/A	N/A	
11	Shinde et al. (2012) [25]	60	M	Headache, seizure, irritability (6months), bilateral abducens	Multifocal: right putamen, left hippocampus, optic nerves and	Wegener's granulomatosis, tuberculosis,	20 (putamen)	Autopsy	T1 iso-hypo T2 hyper T1 CE: hen		yes	Died, never had surgery		

				and facial palsy	leptomeninges	multicentric glioma, meningeosis carcinomatosa							
12	Conner et al. (2012) [7]	42	M	Headache (8 years)	Right subtentorial	Cystic neoplasm, cavernous malformation	12-16	GTR	T2 hyper T1CE.: nhen		yes	no	23	Cavernous (minor-venous-type component)
13		36	M	headache (8 years)	Cerebral falx	Meningeoma	25	STR		hen	yes	no	26	Cavernous (minor-venous-type component)
14	Zhou et al. (2013) [32]	62	M	Seizure (acute onset)	Temporal lobe	Meningeoma	37	GTR	T1 iso T2 hyper T1CE: enhancing		yes	no	7	Venous subtype
15	Li, D. et al. (2014) [20]	23	F	Amenorrhea, visual deficit (8 years)	Cavernous sinus/ clivus/ sellar	Cavernous hemangioma	77	STR	T1 hypo T2 hyper T1-CE: nhen	Hyperdense	yes	no	3	
16		62	M	Disturbance of consciousness,	Right temporal lobe	Meningeoma	35	GTR	T1 iso T2 iso T1-CE: hen	Hyperdense	yes	N/A	N/A	Cavernous

				hypophrasia (2 months)									
17	Lescher et al. (2014) [18]	40	M	Headache, seizure (2 years)	Cerebral falx	Meningeoma, hemangiopericytoma, metastasis	N/A	GTR	T1 iso T2 hyper T1CE: PE		yes	N/A	N/A	Cavernous
18	Sun et al. (2014)[26]	51	F	Visual deficit, bilateral temporal hemianopsia (2 months)	Cavernous sinus, sellar	non-functional pituitary tumor	30	GTR	T1 iso T2 FLAIR hyper T1CE: PE		yes	Died of postoperative complication (pseudoaneurysm of the left internal carotid artery)		
19		49	M	Paraparesis, gait disturbance, nausea, dizziness (one year),	Subtentorial	Hemangioblastoma, Meningeoma	57	STR	T1 hypo T2 hyper T2 FLAIR hyper T1CE: PE		yes	no	12	

20		77	M	Headache (5 months)	Left temporal skull vault invading inward to temporal lobe	Diploic angioma, meningeoma, fibrosarcoma, aneurysmal bone cyst	39	GTR	T1 iso T2 hyper T2 FLAIR hyper T1CE: hen		yes	no	12	
21	Teranishi et al. (2014) [27]	52	F	Right eye discomfort (6 months)	Right cavernous sinus	Trigeminal/ oculomotor nerve schwannoma	23	GTR	T1 iso T2 hyper T1CE: PE		yes	no	2 weeks	Cavernous
22	He et al. (2014) [13]	46	F	Headache (1 year), oculomotor nerve palsy (4 months)	Cavernous sinus	Cavernous sinus hemangioma	20	GTR	T2 hyper		yes	no	72	Cavernous
23		57	M	Headache (10 year), ptosis, diplopia, vision loss (3 months)	Cavernous sinus	Meningeoma	30	GTR	T2 hyper		yes	no	57	Cavernous
24		48	F	Headache (7 years), oculomotor nerve paresis (1 year)	Cavernous sinus	Chordoma	30	GTR	T2 hyper		yes	no	47	Cavernous
25		35	F	Headache (1 year), diplopia, decreased	Cavernous sinus	Angioleiomyoma	20	GTR	T1 hypo T2 hyper	Hyperdense	yes	no	14	Cavernous

				vision (1 week)					T1CE: PE					
26	Xiaofeng et al. (2016) [28]	36	M	Headache, abducens nerve palsy, nausea (2 months)	Cavernous sinus	N/A	60	STR + Radiosurgery	T1 hypo T2 FLAIR hyper T1CE: nhen	Hypo/iso dense	yes	no	20	Cavernous
27	Delgado-Fernandez et al. (2016)[8]	43	M	Hearing loss (6 months)	Subtentorial	Meningeoma	15	GTR	T1 iso T2 hyper T1CE: PE	Hyperdense + enhancing	yes	no	24	Solid
28	Calle et al. (2016) [3]	43	M	Syncope (acute onset)	Cerebral falx	Meningeoma, (aneurysm with CT only)	16	GTR	T1 iso T2 hyper T1-CE:hen	hyperdense	yes	N/A	N/A	Solid
29	Li, CB et al. (2018) [19]	42	M	Headache, vertigo, tinnitus (18 days)	CPA	Meningeoma	31	GTR	N/A	hyperdense	yes	no	37	Cavernous
30		43	M	Incidental finding (1 month)	Right tentorial	Meningeoma	29	GTR	T1 hypo T2 hyper T1-CE: nhen		yes	no	29	Cavernous
31		58	M	Incidental finding	Right parietal lobe		26	GTR	T1 hypo T2 hyper		yes	no	47	Cavernous

									T1-CE: nhen					
32		48	M	Diplopia (24 months)	Cavernous sinus		29	GTR	T1 hypo T2 hyper T1-CE: PE		yes	no	46	Cavernous
33		41	M	Abducens nerve palsy (1 year)	Cavernous sinus	Cavernoma	29	GTR	T1 hypo T2 hyper T1-CE: PE		yes	no	8	Cavernous type
34		47	F	Visual deficit (5 years)	Cavernous sinus	Cavernous hemangioma	31	GTR	T1 hypo T2 hyper T1-CE: nhen		yes	no	34	Cavernous type
35		58	M	Visual deficit (1 year)	Sellar tuberculum		10	STR	T1 hypo T2 hyper T1-CE: nhen		yes	no	8	Cavernous type
36		53	M	Incidental finding	Cerebral falx		30	GTR	T1 hypo T2 hyper		yes	no	5	Cavernous

									T1-CE: nhen					
37	Altieri et al. (2019) [19]	37	M	Incidental finding	Tentorial	Meningeoma/Metastasis	39	GTR	T1 hypo T2 hyper T1-CE: PE	hyperdense	yes	N/A	N/A	N/A
38	Ding et al. (2020) [9]	35	F	Tetraparesis, facial paralysis (1 year)	Lateral ventricle	Meningeoma	74	GTR	T1 hypo T2 hyper T1-CE: nhen		yes	no	5	N/A
39	Chen et al. (2020) [4]	54	F	Headache (2 months), seizure	Right temporal lobe	Meningeoma	13	GTR	T1 hypo T2 hyper T1-CE: PE		yes	no	6	N/A
40	Zhang et al. 2020 [31]	15	M	Frontal headache, rhinorrhea, nose obstruction (2 years)	Right frontal cranial base with intra- and extracranial portion	N/A	30	GTR	T1 hypo T2 hyper T1-CE: PE	hyperdense	yes	no	24	N/A
41	Rubiu et al 2022 [23]	60	F	RIght hemiparesis, ataxia (9 months)	Left tentorial edge	Metastasis	31	STR	T1 hypo T2 hypo		yes	yes	5	

									FLAIR hyper T1CE: nhen					
42	Zhang et al 2023 [34]	15	M	Right nose obstruction, rhinorrhea, frontal headaches	Right frontal cranial base with intra- and extracranial portion		30	GTR	T1 hypo T2 hyper T1CE: PE	hyperdense	yes	no	24	

IAM = internal auditory meatus, CPA = cerebellopontine angle, T1 = MRI T1 sequence, T2 = MRI T2 sequence, T1 CE = T1 with contrast enhancement, hen=homogenously enhancing, nhen= inhomogenous enhancement, enhancing= not specified, if homogenous or inhomogenous PE = progressive enhancement, GTR = Gross total resection, STR = subtotal resection, N/A = not available
